# OPT3 Is a Component of the Iron-Signaling Network between Leaves and Roots and Misregulation of *OPT3* Leads to an Over-Accumulation of Cadmium in Seeds

**DOI:** 10.1093/mp/ssu067

**Published:** 2014-05-31

**Authors:** David G. Mendoza-Cózatl, Qingqing Xie, Garo Z. Akmakjian, Timothy O. Jobe, Ami Patel, Minviluz G. Stacey, Lihui Song, Dustin Wayne Demoin, Silvia S. Jurisson, Gary Stacey, Julian I. Schroeder

**Affiliations:** ^a^Division of Biological Sciences, Cell and Developmental Biology Section and Center for Food and Fuel for the 21st Century, University of California, San Diego, La Jolla, CA 92093, USA; ^b^Division of Plant Sciences, C.S. Bond Life Sciences Center, University of Missouri, Columbia, MO 65211, USA; ^c^Department of Chemistry, College of Chemistry and Chemical Engineering, Xiamen University, Xiamen, Fujian, 361005, China; ^d^ Present address: Laboratory for Infectious Disease Research, University of Missouri, Columbia, MO 65211, USA; ^e^Department of Chemistry, University of Missouri, Columbia, MO 65211, USA; ^f^Department of Biochemistry, C.S. Bond Life Sciences Center, University of Missouri, Columbia, MO 65211, USA

**Keywords:** phloem transport, seed loading, metal homeostasis, iron deficiency, ionomics.

## Abstract

Plants and seeds are the main dietary sources of zinc, iron, manganese, and copper, but are also the main entry point for toxic elements such as cadmium into the food chain. We report here that an *Arabidopsis* oligopeptide transporter mutant, *opt3-2*, over-accumulates cadmium (Cd) in seeds and roots but, unexpectedly, under-accumulates Cd in leaves. The cadmium distribution in *opt3-2* differs from iron, zinc, and manganese, suggesting a metal-specific mechanism for metal partitioning within the plant. The *opt3-2* mutant constitutively up-regulates the Fe/Zn/Cd transporter *IRT1* and *FRO2* in roots, indicative of an iron-deficiency response. No genetic mutants that impair the shoot-to-root signaling of iron status in leaves have been identified. Interestingly, shoot-specific expression of *OPT3* rescues the Cd sensitivity and complements the aberrant expression of *IRT1* in *opt3-2* roots, suggesting that OPT3 is required to relay the iron status from leaves to roots. *OPT3* expression was found in the vasculature with preferential expression in the phloem at the plasma membrane. Using radioisotope experiments, we found that mobilization of Fe from leaves is severely affected in *opt3-2*, suggesting that Fe mobilization out of leaves is required for proper trace-metal homeostasis. When expressed in yeast, OPT3 does not localize to the plasma membrane, precluding the identification of the OPT3 substrate. Our *in planta* results show that OPT3 is important for leaf phloem-loading of iron and plays a key role regulating Fe, Zn, and Cd distribution within the plant. Furthermore, ferric chelate reductase activity analyses provide evidence that iron is not the sole signal transferred from leaves to roots in leaf iron status signaling.

## SUMMARY

Long-distance communication between leaves and roots are key to properly regulate the uptake of trace metals from the soil. The molecular basis of this shoot-to-root signaling is currently unknown. In this manuscript, we describe the role of OPT3 in the shoot-to-root signaling of the iron status in *Arabidopsis*. We also show that reduced expression of *OPT3* induces an over-accumulation of the toxic metal cadmium, but not other metals, in seeds.

## INTRODUCTION

Heavy metals such as iron (Fe), zinc (Zn), copper (Cu), and manganese (Mn) are essential micronutrients for all organisms, acting as co-factors in a variety of biological processes. These heavy metals are extremely reactive and can become toxic at high concentrations; therefore, the intracellular concentration of these essential metals must be tightly regulated (Palmer and Guerinot, 2009; Mendoza-Cozatl et al., 2011). Other heavy metals such as cadmium (Cd), lead, mercury, and the metalloid arsenic (As) do not have biological functions in plants and are toxic even in trace amounts, disrupting several biochemical activities by displacing essential metals from their respective binding sites (Clemens et al., 1998; Mendoza-Cozatl et al., 2011). In humans, Cd exposure has been linked to cancer in the kidneys, lungs, and prostate, and severe Cd poisonings can result in neurological disorders and pulmonary and renal failure (Hendrick, 1996; Il’yasova and Schwartz, 2005; Nawrot et al., 2006). While occupational exposure and tobacco products are associated with a high risk of Cd poisoning, consumption of contaminated plant-based foods represents the major source of Cd exposure in the general public (Franz et al., 2008; Satarug et al., 2010). Many cases of widespread cadmium poisonings have been attributed to consumption of contaminated seeds in Thailand, China, Japan, and Australia (McLaughlin et al., 1997; Clemens et al., 2013). However, the molecular mechanisms and genes mediating the loading of both essential and non-essential heavy metals into seeds remain largely unknown.

Metal accumulation and distribution in plants consist of several mechanisms, including: (1) metal uptake into roots, (2) xylem-loading and transport to the shoot, and (3) phloem-mediated redistribution of metals from mature leaves to sink tissues, including younger leaves, roots, and seeds (reviewed in Mendoza-Cozatl et al., 2011; Palmer and Guerinot, 2009; Verbruggen et al., 2009). Cadmium enters the root through the Fe transporter IRT1, which shows broad substrate specificity towards divalent metals including Fe^2+^, Zn^2+^, Mn^2+^, and Cd^2+^ (Eide et al., 1996; Rogers et al., 2000). Once inside the cell, metals bind to different ligands, according to specific affinities, and these metal–ligand complexes can be stored in different cellular compartments or distributed to other tissues through the vasculature (Verbruggen et al., 2009; Mendoza-Cozatl et al., 2011).

Because of the broad substrate specificity of IRT1 for divalent metals, transcriptional regulation of the Fe-deficiency response, including up-regulation of *IRT1*, will also have an impact on the uptake of non-essential heavy metals such as Cd. In plants, the root iron-deficiency response is regulated by local signals within the root and also by systemic signals originating from leaves (Vert et al., 2003; Walker and Connolly, 2008; Hindt and Guerinot, 2012). Two major transcriptional networks have been identified to mediate the Fe-deficiency response at the root level in *Arabidopsis*: the FIT network and the POPEYE network (Walker and Connolly, 2008; Long et al., 2010; Hindt and Guerinot, 2012). The components of the systemic shoot-to-root Fe signaling on the other hand remain largely unknown. The identification of mutants showing a constitutive Fe-deficiency response even when Fe is supplied in sufficient amounts plus experiments where the constitutive root response is restored by foliar application of Fe suggest that mobile Fe (likely through the phloem) is required for proper shoot-to-root signaling (Vert et al., 2003; Garcia et al., 2013). However, the transporters, ligands, and the chemical speciation of the putative phloem-mobile molecule mediating the systemic Fe signaling have not yet been clearly identified.

Here, we report that *opt3-2*, an *Arabidopsis* mutant carrying an insertion in the 5’ UTR of the oligopeptide transporter gene *OPT3* (Stacey et al., 2008), over-accumulates significant levels of Cd in seeds. We present evidence suggesting that this Cd over-accumulation may be the result of an enhanced transport of Cd through the plant, making *opt3-2* a suitable background for studying long-distance transport of non-essential heavy metals. We further show that OPT3 is targeted to the plasma membrane and is preferentially expressed in the phloem. The Fe/Zn/Mn uptake transporter *IRT1* and other iron-starvation-induced genes are constitutively up-regulated in *opt3-*2. Interestingly, shoot-specific expression of *OPT3* restores metal homeostasis and *IRT1* up-regulation in roots showing that OPT3 is the first identified molecular component of the network transferring information on the iron status from leaves to roots. Moreover, Fe mobilization between leaves is impaired in *opt3-2*, suggesting that OPT3 mediates the movement of Fe out of the leaves, and this transport is required for proper communication between leaves and roots and maintenance of the trace-metal homeostasis in *Arabidopsis*. Understanding phloem-mediated signaling, transport, and seed-loading mechanisms of both essential and non-essential heavy metals will help to develop strategies for excluding toxic metals from seeds and enhance the nutritional value of grains and plant-based products.

## RESULTS

### *opt3-2* Over-Accumulates Cd in Seeds and Shows an Altered Cd Partitioning within Plant Tissues

**Figure 1:**
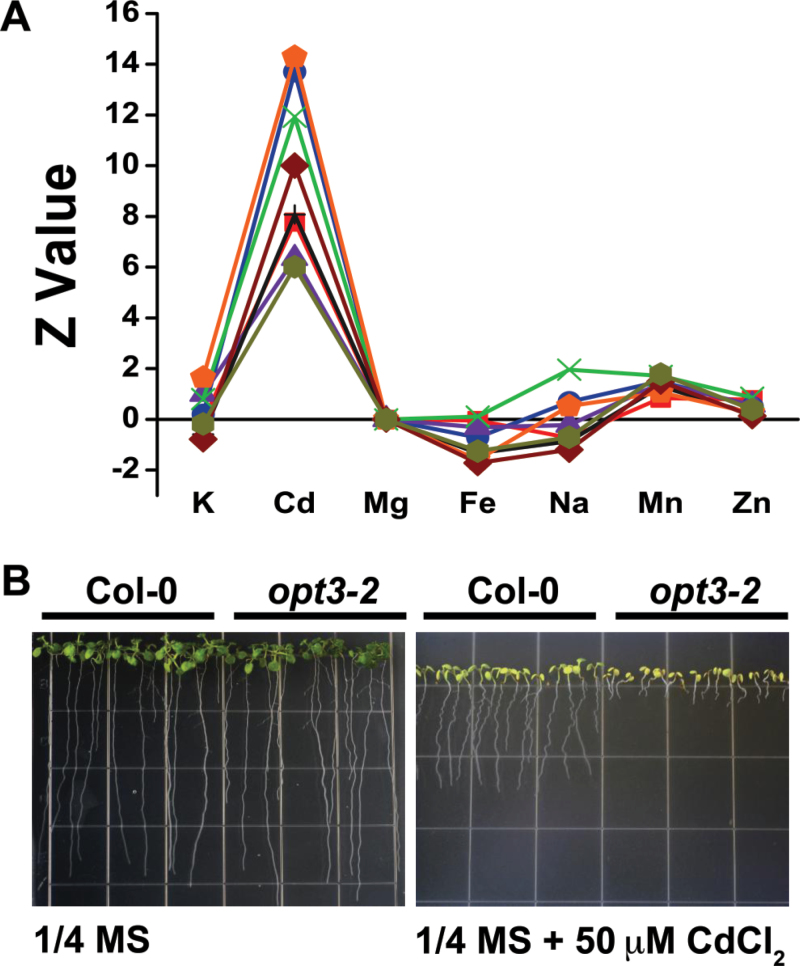
*opt3-2* Over-Accumulates Cd in Seeds and Is Cd-Hypersensitive. **(A)** Ionomic profile of *opt3-2* seeds grown on soil supplemented with heavy metals. Metal concentrations were determined by ICP–OES, normalized against Mg, and plotted as standard deviation from the wild-type mean (*Z*-value) (Lahner et al., 2003). Each line represents seeds from independent plants grown on heavy metal-laden soil. *Z*-values are considered significant when |z| > 1.96 (*p* < 0.05). **(B)** *opt3-2* seedlings are hypersensitive to Cd. Wild-type and *opt3-2* seeds were grown on ¼ MS media with or without 50 μM CdCl_2_ for 2 weeks_._

**Figure 2:**
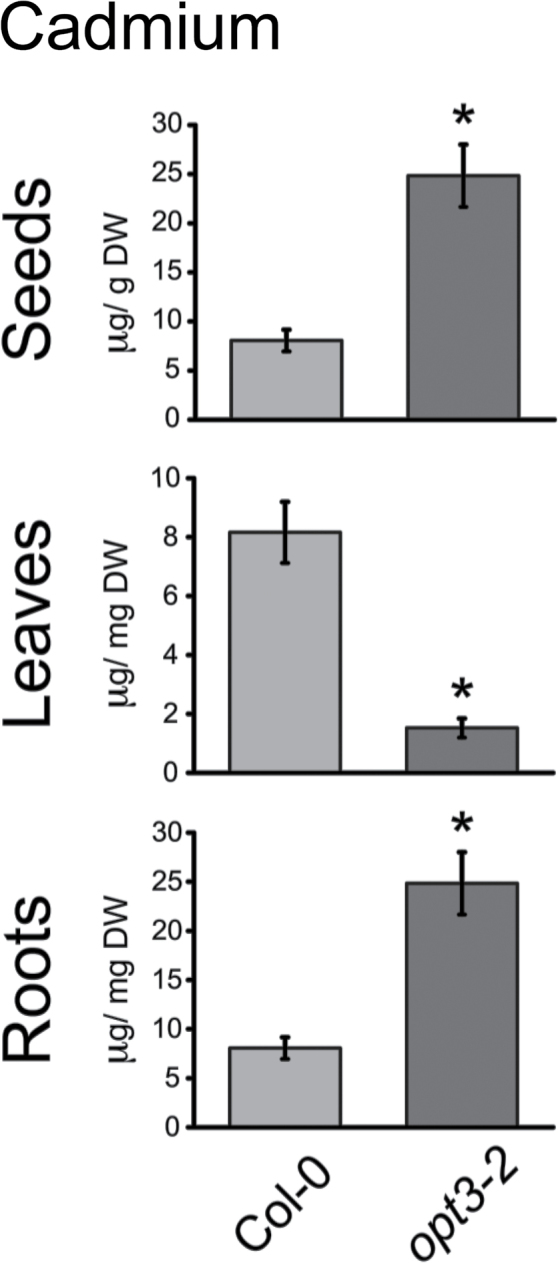
Cadmium Distribution between Tissues Is Altered in *opt3-2* Plants. Cd concentration was measured in roots (*n = 5*) and rosette leaves (*n =* 10) of 6-week-old hydroponically grown plants exposed to 20 μM CdCl_2_ for 72h and dried seeds of plants (*n =* 18) grown on soil containing a defined content of heavy metals (Lahner et al., 2003). Data represent mean ± SE (* *p* < 0.05).

Members of the *Arabidopsis* oligopeptide transporter family (OPT) have been shown to mediate the transport of a broad spectrum of peptides (Koh et al., 2002; Pike et al., 2009). Glutathione (GSH) and phytochelatins are peptides that mediate tolerance and long-distance transport of heavy metals (Mendoza-Cozatl et al., 2008, 2011); therefore, we screened mutants in the *Arabidopsis* OPT family for differential accumulation of Cd in seeds. A mutant of the *Arabidopsis OPT3* gene, *opt3-2*, showed the strongest over-accumulation of Cd in seeds (Figure 1A). To test whether this Cd over-accumulation had an effect on seedling growth, assays were performed on plates in the presence and absence of Cd. Figure 1B shows that *opt3-2* is hypersensitive to Cd when grown on medium containing 50 μM CdCl_2_. To determine whether the increased Cd concentration in *opt3-2* seeds was due to a systemic over-accumulation of Cd throughout the plant, *opt3-2* seedlings were grown hydroponically for 6 weeks, exposed to 20 μM CdCl_2_ for 72h and the metal concentration of roots and leaves was measured by ICP–OES (Figure 2). The roots of *opt3-2* over-accumulated Cd compared to wild-type; however, unexpectedly, Cd concentrations in leaves were almost five-fold less than those of wild-type plants (Figure 2). Conversely, seeds of *opt3-2* plants show a large increase in Cd levels compared to wild-type seeds (Figure 2).

### Cadmium Distribution in *opt3-2* Shoots Is Different from Essential Metals

**Figure 3:**
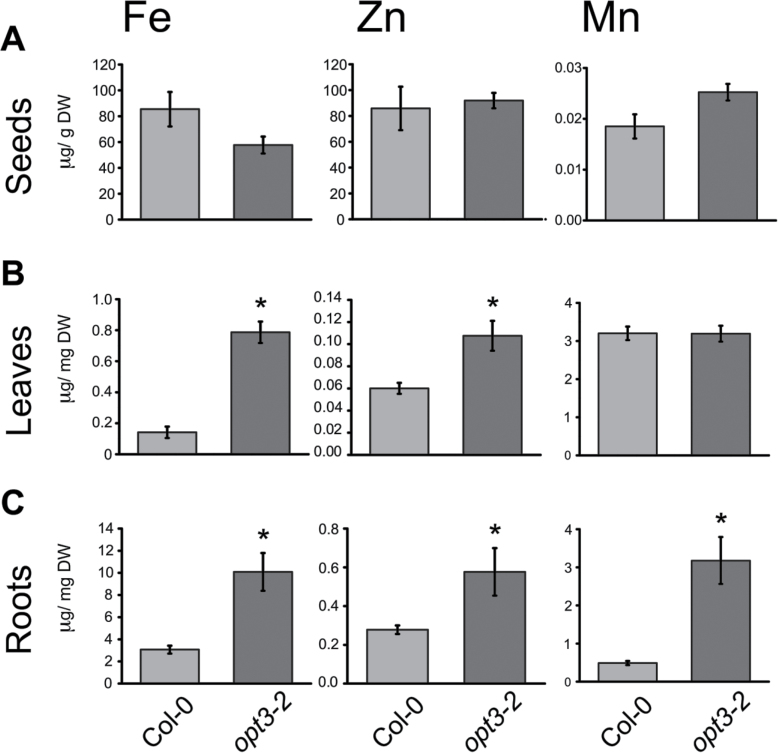
The Distribution of Iron, Zinc, and Manganese Is Different from Cd in *opt3-2*. Metal concentration in roots, leaves, and seeds was determined as in Figures 1 and 2. **(A)** Concentration of Fe, Zn, and Mn in *opt3-2* seeds was similar to wild-type (*n* = 18). **(B)** In leaves, only Zn and Fe were over-accumulated while Mn concentration was unaffected (*n = 10*). **(C)** In roots, *opt3-2* plants exhibited over-accumulation of Zn, Fe, and Mn compared to wild-type plants (*n* = 5). Data represent mean ± SE (* *p* < 0.05).

To determine whether the altered distribution of Cd in *opt3-2* correlated with the distribution of essential metals in plant tissues, the levels of Zn, Fe, and Mn in *opt3-2* were also measured and compared to wild-type plants (Figure 3). No dramatic differences in the concentration of Zn and Mn in seeds were found between wild-type and *opt3-2* (Figure 3A). However, in contrast to Cd accumulation, *opt3-2* over-accumulated significant levels of Zn and Fe in leaves compared to wild-type (Figure 3B). In roots, the concentration of Fe, Zn, and Mn was increased in *opt3-2* compared to wild-type (Figure 3C). The different distribution of Cd in aerial parts of the plants (leaves and seeds) (Figure 2) suggests that the mechanisms mediating accumulation of metals in *opt3-2* leaves is different for Cd compared to the essential metals Fe, Zn, and Mn.

### Ectopically Expressed OPT3 Complements opt3-2

**Figure 4:**
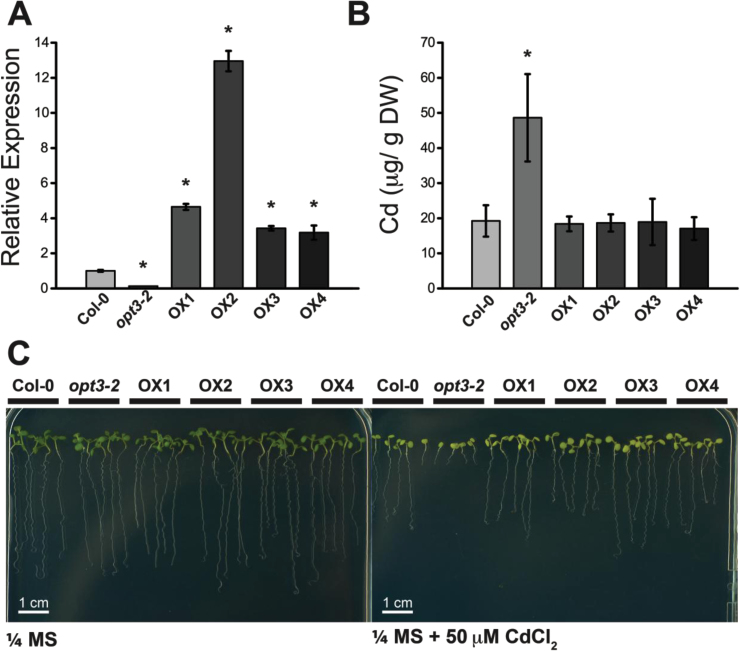
Ectopic Overexpression of *OPT3* in *opt3-2* Reduces Cadmium Concentration in Seeds and Rescues the Seedling Hypersensitivity to Cd. **(A)** Relative *OPT3* expression levels of four representative *35S*_pro_:*OPT3* overexpression lines. Wild-type, *opt3-2*, and four *OPT3* overexpression lines were grown on ¼ MS for 2 weeks, and *OPT3* expression was determined by qPCR and normalized against wild-type *OPT3* expression levels. Data represent mean ± SE (*n* = 3). **(B)** *OPT3* overexpression reduces the over-accumulation of Cd in seeds and **(C)** the Cd hypersensitivity of *opt3-2* seedlings. Wild-type, *opt3-2*, and four complemented lines were grown on ¼ MS with or without 50 μM CdCl_2_ for 2 weeks. Data represent mean ± SE (*n =* 6; * *p* < 0.05).

To evaluate whether the observed metal accumulation phenotypes were due to impaired expression of the *OPT3* gene, the coding sequence of *OPT3* was expressed in *opt3-2* under the control of the *Cauliflower mosaic virus* (*CaMV*) *35S* promoter. Overexpression of *OPT3* in four independent lines was confirmed by qPCR (Figure 4A). *opt3-2* contains a T-DNA insertion in the 5’ UTR of *OPT3* (Stacey et al., 2008). Therefore, the residual OPT3 transcript observed in *opt3-2* is expected in this knockdown line. OPT3 complementation lines were grown on heavy metal-containing soil, and the metal concentration of their seeds was determined by ICP–OES. Cd accumulation in seeds of the four complemented lines was reduced to wild-type levels (Figure 4B). Overexpression also rescued the seedling sensitivity of *opt3-2* to Cd (Figure 4C), indicating that ectopic expression of OPT3 is sufficient to complement the sensitivity and metal accumulation phenotypes of *opt3-2*.

### OPT3 Is a Plasma Membrane Transporter Preferentially Expressed in the Phloem

**Figure 5:**
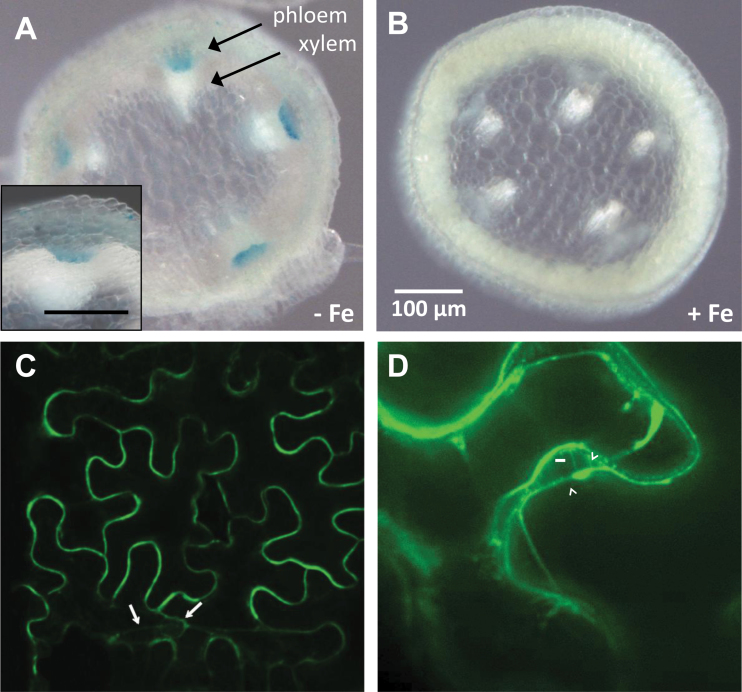
OPT3 Is a Plasma Membrane Transporter Expressed in the Phloem. **(A, B)** GUS staining was performed in fixed and sectioned stems of *OPT3*_pro_:*GUS* plants under (A) iron-deficient conditions and (B) Fe-sufficient conditions. **(C)** OPT3 localizes to the plasma membrane. *N. benthamiana* epidermal cells were infiltrated with *Agrobacterium* carrying *35S*_pro_:*YFP–OPT3* and imaged using confocal microscopy 3 d later. Fluorescence in the cell perimeter is indicative of plasma membrane localization. ER fluorescence is also present (arrows) surrounding the nucleus and as strands traversing the cytoplasm. **(D)** Leaves of *N. benthamiana* epidermal cells transiently expressing *YFP–OPT3* as in the panel were plasmolyzed with 4% NaCl. Note the Hechtian strands (arrows) connecting the cell wall to the plasmolyzed protoplast (double arrowheads), indicative of plasma membrane localization.

Previous GUS staining experiments have shown that OPT3 is expressed throughout the vasculature; however, localization at a higher resolution has not been evaluated (Stacey et al., 2006). To identify where in the vasculature *OPT3* is preferentially expressed, β-glucuronidase (GUS) was expressed under the control of the native *OPT3* promoter. Under standard growth conditions, GUS staining was negligible; however, under Fe-limiting conditions (under which *OPT3* expression is induced), staining was clearly observed in the phloem, but not in the pith or endodermis (Figure 5A and 5B). Consistent with our findings, cell-type-specific microarray data sets show the highest intensity values of *OPT3* in the phloem, comparable to the phloem sucrose transporter *SUC2* (Supplemental Figure 1) (Mustroph et al., 2009). Thus, two independent approaches show preferential expression of *OPT3* in the phloem. To gain insight into the subcellular localization of OPT3, an N-terminal YFP–OPT3 translational fusion was infiltrated into *Nicotiana benthamiana* leaves. Fluorescence was detected along the cell periphery, indicative of plasma membrane localization (Figure 5C). A weaker perinuclear fluorescence and transvacuolar strands were also observed in some cells (see arrows in Figure 5C), indicating that a fraction of the YFP–OPT3 localizes to the endoplasmic reticulum (ER). The ER fluorescence pattern, however, was not present in all cells. Furthermore, Hechtian strands were clearly present connecting the cell wall to the plasma membrane of plasmolyzed leaf cells (Figure 5D). A previous large-scale proteomics study in *Arabidopsis* also found OPT3 at the plasma membrane (Li et al., 2012). These results suggest that OPT3 is a plasma membrane transporter preferentially expressed in the phloem.

### Shoot-Specific Expression of OPT3 Is Sufficient to Restore Metal Homeostasis

**Figure 6:**
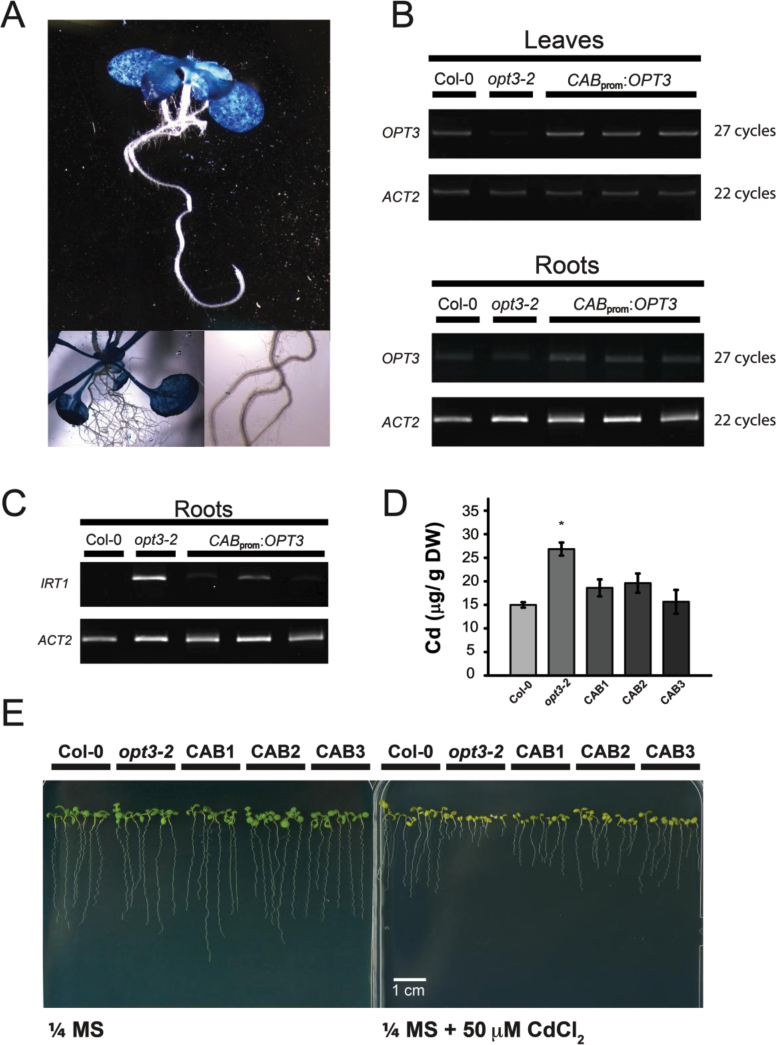
Shoot-Specific Expression of *OPT3* Is Sufficient to Complement the Fe-Deficiency Response in *opt3-2* Roots. **(A)** *CAB2*_pro_ is preferentially active in shoots and is not active in roots. GUS staining in a whole seedling expressing *CAB2*_pro_:*GUS* is evident only in shoots. **(B)** RT–PCR confirmed the shoot specificity of *CAB2*_pro_:*OPT3.* Wild-type, *opt3-2*, and three independent *CAB2*_pro_:*OPT3* lines were grown vertically on ¼ MS plates for 2 weeks, and cDNA was prepared separately from root and leaf RNA. *OPT3* expression was determined in roots and shoots of wild-type, *opt3-2*, and three independent *CAB2*_pro_:*OPT3* lines. *ACT2* was used a loading control, and the number of PCR cycles is shown to the right of each gel image. Note that complete knockout of *OPT3* causes embryo lethality (Stacey et al., 2002), and *opt3-2* shows reduced expression of *OPT3* transcript. **(C)** *CAB2*_pro_:*OPT3* successfully restores regulation of *IRT1* in *opt3-2*. *IRT1* expression in roots of wild-type, *opt3-2*, and *CAB2*_pro_:*OPT3* was determined by RT–PCR as in panel (A). RT–PCR was performed for 22 cycles, and *ACT2* was used as a loading control. **(D)** *opt3-*2 plants expressing *CAB2*_pro_:*OPT3* accumulate wild-type levels of Cd in seeds. Wild-type, *opt3-2*, and three *CAB2*_pro_:*OPT3* lines were grown on heavy metal-laden soil, and their seed metal concentration was determined by ICP–OES as in Figure 1. Data represent mean ± SE (*n* = 6; * *p* < 0.05). **(E)** *CAB2*_pro_:*OPT3* complements seedling sensitivity to Cd in *opt3-2*. Wild-type, *opt3-2*, and three *CAB2*_pro_:*OPT3* lines were grown on ¼ MS with or without 50 μM CdCl_2_ for 2 weeks.

The *Arabidopsis* mutant *opt3-2* shows a constitutive Fe-deficiency response in roots including the up-regulation of the Fe/Zn/Mn transporter *IRT1* (Stacey et al., 2008). Despite this Fe-deficiency response, Fe sensing in shoots remains intact (Stacey et al., 2008). The molecular mechanisms mediating shoot-to-root signaling of iron status in plants remain largely unknown. The impaired iron sensing in roots but not shoots of *opt3-2*, in conjunction with phloem localization, suggests a possible role of OPT3 in shoot-to-root transport of a signal reporting metal status. To test this hypothesis, the *OPT3* coding sequence was expressed in *opt3-2* under the control of the shoot-specific chlorophyll *a/b* binding protein promoter (*CAB2*_pro_:*OPT3*) (Chen et al., 2006). Shoot specificity of the *CAB2* promoter was determined by GUS staining (Figure 6A). RT–PCR analyses confirmed that *OPT3* is preferentially expressed in the shoots of three independent transgenic lines (Figure 6B). The residual *OPT3* transcript in *opt3-2* roots expressing *CAB2*_pro_:*OPT3* plants is consistent with the knockdown nature of the *opt3-2* allele. Thus, the low level of *OPT3* transcript in roots is not sufficient to properly regulate metal homeostasis in roots (Stacey et al., 2008).

Two of the major phenotypes described in *opt3-2* are the constitutive iron-deficiency response in roots, as illustrated by high *IRT1* expression (Figure 6C), and the over-accumulation of Cd in seeds (Figure 6D). Thus, we tested whether shoot-specific expression of *OPT3* was able to complement both phenotypes. As shown by RT–PCR, *IRT1* transcript levels were greatly reduced in the roots of *CAB2*_pro_:*OPT3*-expressing plants compared to the *opt3-2* mutant (Figure 6D). These results show that shoot-specific expression of *OPT3* is sufficient for proper regulation of metal homeostasis, including communication between leaves and roots. Furthermore, the Cd accumulation in *CAB2*_pro_:*OPT3* seeds was reduced to wild-type levels (Figure 6D). Seedling hypersensitivity to Cd was also rescued in the three independent *CAB2*_pro_:*OPT3*-expressing lines (Figure 6E). Collectively, these results demonstrate that shoot-specific *OPT3* expression is sufficient to complement *opt3-2* root phenotypes, suggesting that OPT3 may mediate the long-distance transport of a signaling molecule from leaves to relay information about metal status, thus contributing to whole-plant metal homeostasis.

### Leaf-to-Leaf Transport of Fe Is Impaired in *opt3-2*

**Figure 7:**
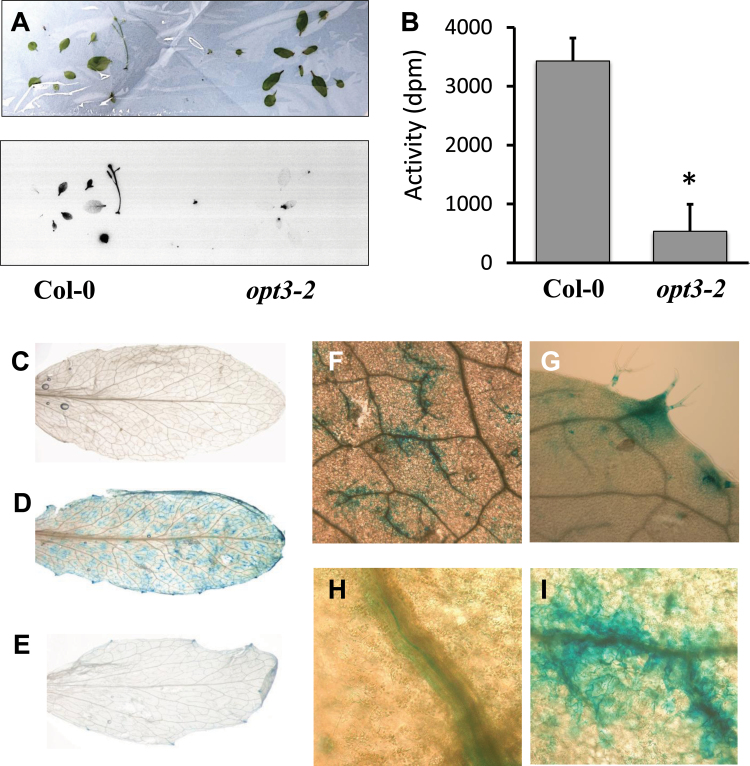
Mobilization of Iron between Leaves Is Impaired in *opt3-2*. **(A)** ^59^Fe was applied to a mature wild-type or *opt3-2* leaf and the distribution of ^59^Fe was monitored after a 12-h incubation period. Lower panel: Signal coming from ^59^Fe was detected in wild-type leaves adjacent to the leaf where the ^59^Fe was applied while only a fraction of the signal was detected in *opt3-2* leaves. **(B)** The specific activity measured in the four adjacent leaves to which the ^59^Fe was originally applied show that the movement of ^59^Fe in *opt3-2* was marginal. Data represent mean ± SE (*n* = 4, * *p* < 0.05). **(C–I)** Visualization of Fe using Perls’ stain shows that, compared to a mature wild-type leaf (C), *opt3-2* contains substantial amounts of stainable Fe (D). (E) Over-accumulation of Fe in *opt3-2* leaves is less evident in younger leaves. Accumulation of Fe in opt3-2 leaves is more evident close to (F) secondary veins, (G) the base of trhichomes, and (H, I) surrounding the vasculature.

To test whether OPT3 functions in the mobilization of Fe or other molecules, we first assessed the capacity of wild-type and *opt3-2* to remobilize Fe from one leaf to other leaves using the radiotracer ^59^Fe. In these experiments, ^59^Fe was loaded into a mature leaf as Fe^2+^ at a slightly acidic pH (pH 6.2) to resemble the apoplastic pH. The addition of ascorbic acid was used to reduce Fe^3+^ to Fe^2+^ and maintain it in the reduced form. Figure 7A shows that Fe can be re-mobilized from one leaf to adjacent leaves in wild-type. In contrast, *opt3-2* shows negligible movement of ^59^Fe between leaves. Figure 7B shows the ^59^Fe activity (dpm) in the four leaves adjacent to the leaf where the ^59^Fe was originally applied. Compared to wild-type, *opt3-2* shows a severe reduction in the quantity of ^59^Fe mobilized from one leaf to the adjacent leaves (Figure 7B), suggesting that OPT3 is required for the reallocation of Fe between plant tissues. In fact, *opt3-2* plants over-accumulate Fe in mature leaves compared to wild-type, as visualized by Perls’ staining (Figure 7C and 7D). Interestingly, over-accumulation of Fe in *opt3-2* occurs only in mature leaves but not in young leaves (Figure 7D and 7E). Moreover, accumulation of Fe in *opt3-2* is more evident at the base of the trichomes and near the vasculature in the minor veins but not in the main vasculature, suggesting that, in *opt3-2*, the reallocation of Fe between leaves is impaired, particularly at advanced stages of leaf development (Figure 7F–7I).

To test whether OPT3 functions as a Fe^2+^ transporter similarly to IRT1, we expressed OPT3 in a yeast strain deficient in Fe^2+^ uptake (*fet3fet4*). As previously shown, IRT1 expression in yeast allows the *fet3fet4* strain to grow on minimal media without the addition of extra Fe (Supplemental Figure 2). OPT3 was unable to rescue the *fet3fet4* strain (Supplemental Figure 2), suggesting that, in yeast, OPT3 does not mediate the uptake of Fe^2+^ like IRT1. Subcellular localization studies, however, show that OPT3–YFP protein fusions do not localize to the plasma membrane in yeast (Supplemental Figure 3) in contrast to *in planta* (Figure 5C). This mislocalization of OPT3 in yeast precluded further characterization of OPT3 using yeast as a heterologous system. Note that, if *fet3fet4* yeast cells were not sufficiently pre-starved of iron, growth of the *fet3fet4* mutant was observed, and therefore long-term starvation of yeast was required for these complementation tests. We attempted complementation with different yeast promoters, starting with the strong GAL promoter (Supplemental Figure 2A). Using the phosphoglycerate kinase (PGK) yeast promoter, OPT3 also did not complement the pre-iron-starved f*et3fet4* yeast mutant, consistently with previous studies showing that OPT3 does not complement this yeast mutant (Supplemental Figure 2A). Nevertheless, ^59^Fe re-mobilization studies suggest that OPT3 is essential for the remobilization of Fe within plant tissues; whether this transport occurs as Fe^2+^ or as a Fe-ligand complex remains to be determined.

OPT3 is a member of the oligopeptide transporter family and some members of this family have been found to have broad substrate specificity for peptides of different length and amino acid composition (Osawa et al., 2006; Pike et al., 2009). To test whether OPT3 mediates the long-distance transport of GSH *in planta*, we pursued radiotracer experiments to assess the movement of ^35^S-GSH from one leaf to adjacent leaves (Supplemental Figure 4). No differences were found between wild-type and *opt3-2*, suggesting that OPT3 does not participate in the mobilization of GSH between plant tissues. Interestingly, *opt3-2* rosette leaves from plants exposed to 20 μM CdCl_2_ (in the hydroponic solution) and supplemented with 0.5mM GSH (foliar application) accumulated Cd, but no other metals, to wild-type levels. These results suggest that GSH is required for Cd retention in leaves. On the other hand, GSH supplemented to the roots reduced Cd in the leaves of both *opt3-2* and wild-type plants (Supplemental Figure 5) likely because GSH trapped Cd in roots of both *opt3-2* and wild-type plants (Supplemental Figure 5).

Glutathione has recently been shown to play a critical role in Fe signaling in yeast by stabilizing FeS clusters in the cytosol (Rutherford et al., 2005; Li et al., 2009). In *Arabidopsis*, GSH is also important to maintain proper homeostasis and crosstalk between Zn and Fe metabolism (Shanmugam et al., 2012). To test whether long-distance transport of GSH is important for proper shoot-to-root signaling and homeostasis of trace metals in roots, we measured the constitutive high activity of the root ferric reductase in *opt3-2* after foliar application of GSH (Supplemental Figure 6A). In all cases, including the application of foliar GSH, GSH applied to roots, or foliar application of Fe, the activity of the root ferric reductase remained constitutively high in *opt3-2.* We also tested whether iron applied directly to roots or complexed with GSH, citrate, or nicotianamine is sufficient to repress the high activity of the Fe chelate reductase in *opt3-2* roots. Supplemental Figure 6B shows that iron alone (Fe^2+^), or in complex with GSH, nicotianamine, or citrate, cannot down-regulate the constitutive iron-deficiency response in *opt3-2* back to wild-type levels.

## DISCUSSION

### *opt3-2* Shows an Altered Phloem-Mediated Cd Distribution

We have identified an *Arabidopsis* mutant, *opt3-2*, that over-accumulates Cd in seeds and roots (Figure 1) but, unexpectedly, under-accumulates Cd in leaves (Figure 2). Cadmium distribution throughout the plant is an orchestrated process dictated by root uptake, root-to-shoot translocation through the xylem, and redistribution of Cd from leaves to sink tissues (i.e. seeds, younger leaves, and roots) via the phloem. *opt3-2* displays constitutive up-regulation of *IRT1*, a root transporter with broad specificity for heavy metals including Cd (Eide et al., 1996; Rogers et al., 2000). Over-accumulation of Cd, Zn, Fe, and Mn in roots (Figures 2 and 3) may be explained by the constitutively high expression of *IRT1*. However, under-accumulation of Cd in leaves and over-accumulation of Cd in seeds, which is different from essential metals (Figures 1 and 2), is inconsistent with the high expression of *IRT1* (Stacey et al., 2008). Nutrients, water, and heavy metals are mobilized from leaves into seeds through the phloem (Turgeon and Wolf, 2009). Accumulation of metabolites in sink tissues (roots and seeds) and under-accumulation in source tissues (leaves) is best described as an increased redistribution process, likely through the phloem (Nour-Eldin et al., 2012). Notably, of the analyzed metals, only Cd under-accumulates in leaves (Figure 2). These results suggest that, in contrast to the broad specificity of heavy metal uptake at the root level, metal-specific mechanisms mediate the remobilization of heavy metals from leaves to sink tissues.

In addition to the altered distribution of heavy metals within the plant leading to over-accumulation of Cd in seeds, *opt3-2* also shows hypersensitivity to Cd at the seedling stage (Figures 1–3). Both the increased accumulation of Cd in seeds and the Cd hypersensitivity of seedling growth are restored to wild-type levels by ectopically expressing *OPT3*, demonstrating that the altered redistribution of Cd through the plant is the result of the reduced expression of *OPT3* in *opt3-2* (Figure 4).

### OPT3 Mediates Shoot-to-Root Signaling of Iron Status

The *opt3-2* mutant displays a constitutive iron-deficiency response in roots, while the leaves properly respond to iron levels as indicated by wild-type levels of ferritin expression (Stacey et al., 2008), suggesting that the iron status response is mainly disrupted in roots. In plants, the root iron-deficiency response is regulated by local signals within the root and also by unknown systemic signals originating from aerial tissues (Vert et al., 2003; Hindt and Guerinot, 2012). OPT3 is a plasma membrane transporter preferentially expressed in phloem cells during iron starvation (Figure 5). Cell-specific microarrays (Mustroph et al., 2009) (Supplemental Figure 1) and *OPT3*_pro_:*GUS* analysis under Fe-limiting conditions (Figure 5A) show preferential expression of OPT3 in phloem cells, suggesting a role of OPT3 in long-distance transport processes. Notably, shoot-specific expression of *OPT3* (*CAB2*_pro_:*OPT3*) in the *opt3-2* background rescued the constitutively high expression of IRT1 in roots, the seed Cd over-accumulation phenotype, and the seedling sensitivity to Cd (Figure 6). These results suggest that the impaired metal homeostasis in *opt3-2* roots is caused by a disruption of the shoot-to-root signaling of the leaf metal status. Thus, OPT3 is the first shoot-expressed gene required for proper communication from leaves to roots to maintain metal homeostasis at the whole-plant level.

Several *Arabidopsis* and tomato mutants displaying an Fe-deficiency response in roots can be rescued by foliar application of Fe (Garcia et al., 2013); these experiments suggest that shoot-to-root Fe signaling plays an important role in Fe homeostasis (Garcia et al., 2013) which in turn could also impact the uptake and accumulation of other transition metals such as Zn, Mn, and Cd, as seen in *opt3-2* (Figures 1 and 3). Foliar application of Fe does not repress the Fe-deficiency response in *opt3-2* roots to wild-type levels (Garcia et al., 2013) (Supplemental Figure 6), suggesting that source-to-sink transport of Fe, or a molecule mediating Fe signaling, is impaired in *opt3-2*. Radiotracer experiments using ^59^Fe demonstrate that the movement of Fe between leaves is impaired in *opt3-2* (Figure 7A); whether this leaf-to-leaf transport occurs as Fe^2+^ or as an Fe–ligand complex remains to be determined. OPT3 is a member of the oligopeptide transporter family and members of this family have been shown to mediate the transport of a broad range of peptides (Osawa et al., 2006; Pike et al., 2009). *Arabidopsis* OPT3 has also been reported to rescue the ability of yeast mutants defective in Cu and Mn transport to grow on low concentrations of these transition metals (Wintz et al., 2003). However, so far there is no direct evidence to suggest that OPT3 mediates the transport of transition metals, in the ionic form or complexed with a ligand, or whether OPT3 mediates the transport of a ligand that facilitates the uptake and accumulation of transition metals into the cell. In fact, our OPT3 localization experiments in yeast show that OPT3–YFP fusions are unable to transit out of the ER to the plasma membrane (Supplemental Figure 3). This intracellular localization of OPT3 makes it difficult to interpret the ability of yeast strains defective in transition metal transport to grow on minimal media when expressing OPT3.

Glutathione is a small peptide that has also gained recent attention in metal-status signaling via GSH-coordinated intermediaries of the iron–sulfur cluster assembly machinery (Rutherford et al., 2005; Rouhier et al., 2007; Bandyopadhyay et al., 2008). Regulation of GSH levels is also essential for regulating the iron-deficiency response in fungi (Li et al., 2009; Kumar et al., 2011). Our radiotracer experiments using ^35^S-GSH (Supplemental Figure 4) and the ferric reductase assay in *opt3-2* roots (Supplemental Figure 6) show that leaf-to-leaf movement of GSH was unaffected and that foliar application of GSH does not suppress the constitutive Fe-deficiency response in *opt3-2*. These results suggest that shoot-to-root transport of GSH alone has little effect on the long-distance signaling of the Fe status in *Arabidopsis*.

### *opt3-2* as a Model for Long-Distance Cd and Nutrient Transport

Phloem transport plays a key role in delivering nutrients, including metals, to developing seeds (Turgeon and Wolf, 2009; Mendoza-Cozatl et al., 2011). However, the mechanisms of toxic heavy metal loading into seeds are largely unknown. Nicotianamine, GSH, and PCs are the main metal-chelating molecules found in phloem sap (Mendoza-Cozatl et al., 2011). Nicotianamine has been shown to form complexes with Fe, Cu, Zn, and Mn, while GSH and PCs preferentially bind to Cd (Dorčák and Krężel, 2003). The differential partitioning of Cd among roots, leaves, and seeds in *opt3-2* relative to the essential metals Fe, Zn, and Mn suggests that independent mechanisms mediate the partitioning of essential and non-essential metals, likely as specific metal–chelate complexes. Understanding phloem-mediated transport and seed-loading mechanisms of individual metals and metal–ligand complexes will be important to restrict accumulation of toxic metals in seeds while ensuring the accumulation of essential metals.

In summary, we show that *Arabidopsis* OPT3 is expressed in the phloem and functions in the long-distance shoot-to-root signaling of Fe/Zn/Mn status. When OPT3 expression is compromised, there is a misregulation of genes mediating uptake and mobilization of trace metals leading to an over-accumulation of cadmium, but not other metals, in seeds. We further show that mobilization of Fe^2+^ between leaves is impaired in *opt3-2* and that targeted OPT3 expression in leaves is sufficient to restore Fe/Zn/Mn status signaling to roots providing molecular information on shoot-to-root Fe-status signaling. Sensing and regulation of trace-metal homeostasis in plants have been long-standing questions in plant biology and the results presented here offer new insights and avenues to advance our understanding of how essential and non-essential metals are accumulated and distributed within plant tissues.

## METHODS

### Plant Materials and Growth Conditions

Wild-type (Col-0) and *opt3-2* seeds were surface-sterilized, stratified at 4°C for 48h in the dark, and germinated under a 16-h light/8-h dark photoperiod. For Cd sensitivity experiments, ¼ MS plates were supplemented with 50 μM CdCl_2_ and allowed to grow vertically for 14 d.

For metal determination in seeds, 2-week-old seedlings were transferred to Sunshine Basic Mix 2 soil supplemented with heavy metals as described (McDowell et al., 2013). For metal determination in roots and leaves, plants were grown hydroponically as described previously (Chen et al., 2006). Elemental analyses were performed by ICP–OES at the UCSD/Scripps Institution of Oceanography analytical facility using dried rosette leaves, roots, or seeds digested overnight in trace-metal grade 70% HNO_3_ as described previously (Chen et al., 2006).

### Plasmid Construction

All primers used for PCR amplification for cloning are listed in Supplemental Table 1. For *OPT3* expression driven by the *CaMV 35S* promoter (*35S*_pro_:*OPT3*), the *OPT3* genomic DNA fragment was amplified using the primers OPT3-A and OPT3-B. The amplified *OPT3* DNA was cloned as an *Avr*II/*Bst*EII fragment into a modified pCambia 1391Z binary vector encoding the *CaMV 35S* promoter derived from pRT101 (Topfer et al., 1987). For confocal localization studies, the *OPT3* coding sequence was amplified from Col-0 cDNA to create pENTR–OPT3 using OPT3-C and OPT3-D, and cloned into pENTR/D-TOPO® (Invitrogen, Carlsbad, CA, USA). The *YFP–OPT3* fusion was obtained by recombining the *OPT3* coding sequence into pH35YG (Nishimura et al., 2010) using LR Clonase II® (Invitrogen, Carlsbad, CA, USA).

For shoot-specific expression, the *OPT3* coding sequence was recombined into a Gateway® compatible pGreenII plasmid (Hellens et al., 2000) containing the *CAB2*_pro_ and the *NOS* terminator (*CAB2*_pro_:GW–*NOS*_ter_). The *CAB2* promoter was amplified from Col-0 genomic DNA using the primers CABP-A and CABP-C, and cloned into the *Kpn*I/*Hin*dIII sites of pGreenII upstream of the Gateway® cassette. For *CAB2*_pro_ GUS staining, the 2-kb promoter fragment was amplified from genomic DNA using CABP-B and CABP-C, inserted into pENTR/D-TOPO®, and recombined into pBGGUS (Kubo et al., 2005). For *OPT3*_pro_:YFP expression studies, a 2-kb fragment upstream of the start codon of *OPT3* was amplified from Col-0 genomic DNA using the primers OPT3P-A and OPT3P-B, and introduced into pDONRZeo® using BP Clonase II® (Invitrogen, Carlsbad, CA, USA). *OPT3*_pro_ was then recombined into a Gateway®-compatible pGreen II plasmid containing the coding sequence of YFP and the *NOS* terminator. For localization of OPT3 in *Saccharomyces cerevisiae*, the N-terminal YFP fusion *YFP–OPT3* was amplified from pH35YG–OPT3 with YFP-A and OPT3-D, cloned into pENTR/D-TOPO®, and inserted into pYES–DEST52 via recombination. The C-terminal YFP fusion *OPT3–YFP* was created by amplifying *OPT3* with OPT3-E and OPT3-F, and amplifying *YFP* with YFP-B and YFP-C. The two PCR fragments were combined with the USER® system (New England Biolabs, MA, USA), inserted into pDONRZeo® using BP Clonase II®, and then recombined into pYES–DEST52.

### Plant Transformation

*A. thaliana* was transformed using the floral dip method (Clough and Bent, 1998), and *N. benthamiana* was Agro-infiltrated as previously described (Wydro et al., 2006). *Agrobacterium tumefaciens* strain GV3101 was used for all transformations, and pSoup was used as the helper plasmid for pGreenII-carrying strains (Hellens et al., 2000). *OPT3*_pro_:*YFP* was transformed into Col-0 plants, and *CAB2*_pro_:*OPT3* and *35S*_pro_:*OPT3* were transformed into the *opt3-2* background.

### Reverse Transcription PCR and Quantitative PCR

For RT–PCR of *CAB2*_pro_:*OPT3* transgenic lines, plants were grown vertically on ¼ MS media for 14 d. Leaves and roots were then separated, and RNA was prepared using the RNEasy Plant Mini Kit® (Qiagen, Hilden, Germany). The RNA was DNase treated and reverse-transcribed with SuperscriptIII® (Invitrogen, Carlsbad, CA, USA). RT–PCR was performed for the indicated number of cycles and normalized against *ACT2* using the primers listed in Supplemental Table 2. RT–PCR was performed for additional cycles to confirm that amplification at the indicated cycles were in the logarithmic phase.

For qPCR analysis of *35S*_pro_:*OPT3* lines, plants were grown on ¼ MS media for 14 d. cDNA was then prepared from RNA of whole seedlings as for RT–PCRs. Transcript abundance was then determined with SYBR® Green (Sigma-Aldrich, St. Louis, MO, USA) using a LightCycler® 1.5 Real-Time PCR System (Roche Diagnostics, Indianapolis, IN, USA). *OPT3* transcript levels were normalized to *ACT2* transcript levels and relative *OPT3* expression levels were determined using the comparative C_t_ method (Schmittgen and Livak, 2008). Primers used for qPCR are listed in Supplemental Table 3.

### Yeast Transformation and Growth

*S. cerevisiae* BY4741 was transformed with pYES–DEST52 plasmids harboring *YFP–OPT3* or *OPT3–YFP* via the lithium acetate method (Schiestl and Gietz, 1989), and transformants were selected on glucose-containing YNB-Ura media (Mäser et al., 2002). Expression of *YFP–OPT3* and *OPT3–YFP* was induced by culturing yeast overnight in YNB-Ura supplemented with 2% galactose and 1% raffinose.

### GUS Staining and Fluorescence Microscopy

For GUS staining, transgenic plants carrying the *OPT3*_pro_:*GUS* fusion were grown on ½ MS medium for 20 d before being transferred to Fe-sufficient or Fe-deficient medium (Yi and Guerinot, 1996) and grown for an additional 10 d. Inflorescence stems were isolated, hand-sectioned, and stained for GUS as previously described (Stacey et al., 2002). *CAB2*_pro_:*GUS* staining was performed on 3-week-old seedlings grown on ¼ MS medium. Staining patterns were observed and documented using a Nikon SMZ1500 stereomicroscope. Fluorescence microscopy was performed on a Nikon TE-200U microscope equipped with a Yokogawa Nipkow spinning disc confocal head and a Roper Cascadell 512b EM CCD camera. YFP was excited with a Chroma HQ480/40 band-pass emission filter. Fluorescence images were captured using Metamorph v.5.0 (Universal Imaging, Sunnyvale, CA, USA) and edited using NIH ImageJ (http://imagej.nih.gov/ij/). For plasmolysis experiments, *N. benthamiana* leaf sections were incubated in 4% NaCl for 15min prior to imaging.

### Leaf-to-Leaf Transport of ^59^Fe and ^35^S-GSH

Plants grown for at least 3 weeks under 16-h/8-h light/dark cycles were used for radiotracer experiments. For ^59^Fe experiments, a fully developed leaf was immersed in a buffer containing 50mM MES (pH 6.2), 15 μM FeCl_3_, 1mM ascorbic acid, and 30 μCi ml^–1^ of ^59^Fe (Perkin Elmer, USA). Wild-type and *opt3-2* leaves were incubated with the radiotracer for 12h before detaching the load leaf, which was placed into a separate 20-ml scintillation vial to determine its specific activity. Following the incubation, the remaining rosette leaves were dissected onto a platform for autoradiography using a phosphorimaging plate (Fujifilm 20 cm × 40 cm phosphorimaging plate, BAS-IP MS 2040, or Fujifilm 20 cm × 25 cm phosphorimaging plate, BAS-IP MS 2025). The phosphorimaging plate was exposed for 2h and then scanned on a Typhoon FLA 9000 (GE Healthcare Lifesciences) using the phosphorimaging settings with 100-μm resolution (approximately 15-min scan). After scanning, the dissected leaves were placed in 20-ml scintillation vials. Opti-Fluor (10 ml, PerkinElmer) was added to each sample and the specific activity was determined using a liquid scintillation counter (TriCarb Liquid Scintillation Counter, PerkinElmer). To reduce the excitation between samples with higher activity, two spaces in the racks were left empty between samples. A background sample was analyzed and its count rate subtracted from each sample.

Remobilization of ^35^S-GSH between leaves was performed as described for ^59^Fe with some modifications. A mature leaf from wild-type or *opt3-2* was immersed in a solution containing 25mM GSH, Tris 50mM (pH 7.5) supplemented with ^35^S-GSH (30 μCi ml^–1^, Perkin Elmer, USA). After a 12-h incubation under continuous light, the immersed leaf was removed and the remaining rosette leaves were dissected and placed on the phosphorimaging plate which was exposed for 5h and then scanned using a Typhoon FLA 9000 (GE Healthcare Lifesciences).

### Perls’ Staining of Ferric Iron

The Perls’ staining method was used as described (Green and Rogers, 2004; Schuler et al., 2012). Mature leaves, young leaves, and siliques were harvested freshly from 7–8-week-old plants and incubated with fixative solution (methanol/chloroform/pure acetic acid, 6:3:1) for 1h at room temperature. After incubation, the fixative solution was removed, and the samples were washed by exchanging distilled water three times. Perls’ staining solution (4% HCl and 4% K-ferrocyanide, 1:1; Green and Rogers, 2004) was added for 1h at room temperature. The reaction was terminated by washing three times with distilled water.

### Cd Treatment Together with Foliar and Root Application of GSH

Plants were grown on soil until right before bolting (5–6 weeks old) and then transferred to 0.5 Hoagland’s nutrient hydroponic solution pH 5.9 (Heeg et al., 2008) for 1 week and exposed to defined treatments for 72h as follows: (A) Cd: 0.5 Hoagland’s nutrient hydroponic solution + 20 μM Cd; (B) Cd+Shoot GSH: same as Cd treatment with (0.5mM GSH + 0.125%Tween20 (added as surfactant) application to leaves; (C) Cd+Root GSH: same as Cd treatment hydroponic media with 0.5mM GSH added to the hydroponic solution. Tissue digestion and elemental concentrations were determined as described above.

### Ferric Reductase Activity Determinations

Plants were grown on soil right until before bolting and then transferred to 0.5 Hoagland’s hydroponic nutrient solution pH 5.9 described above for 1 week and then transferred to Fe-limited hydroponic solution (0.5 Hoagland’s nutrient solution pH 5.9 without Fe-EDTA) for 7 d. Plants were then transferred to the following treatments: –Fe + Foliar Fe: hydroponic nutrient solution without Fe and with (0.05% FeSO4 + 0.125%Tween20 application to leaves; –Fe + Foliar Fe + Shoot GSH: same as –Fe + Foliar Fe, with 0.5mM GSH application to leaves; –Fe + Foliar Fe + Root GSH: same as –Fe + Foliar Fe but adding 0.5mM GSH to the Fe-limited hydroponic solution. For foliar Fe and GSH spray treatments, leaves were sprayed once a day until they were completely moistened. After 5 d of foliar treatment, root ferric reductase activity was determined as previously described (Lucena et al., 2006).

## SUPPLEMENTARY DATA

Supplementary Data are available at *Molecular Plant Online.*

## FUNDING

This research was supported by a National Institute of Environmental Health Sciences (Grant No. P42 ES010337), OPT3 transport analyses were funded by the Chemical Sciences, Geosciences, and Biosciences Division of the Office of Basic Energy Sciences at the US Department of Energy (DE-FG02-03ER15449). Research was supported by a US National Science Foundation Arabidopsis 2010 grant (IOB 0419695 to J.I.S.) and a US National Science Foundation CAREER grant (IOS-1252706 to D.G.M.C.). D.G.M.C. also received support from the University of Missouri Research Board Grant (Project CB000519). ^59^Fe and ^35^S-glutathione experiments were supported by the Department of Energy (Projects for Interrogations of Biological Systems, DE-SC0002040 to S.S J.). T.O.J. was supported by the UCSD-Salk IGERT Plant Systems Biology Interdisciplinary Graduate Training Program (Grant No. 0504645) and D.W.D. by the NIH-NIBIB Training Grant (5 T32 EB004822).

## Supplementary Material

Supplementary Data
