# Supplementary material for: OPT3 Is a Component of the Iron-Signaling Network between Leaves and Roots and Misregulation of OPT3 Leads to an Over-Accumulation of Cadmium in Seeds
Source: Mol Plant. 2014 May 31;7(9):1455–69. doi: 10.1093/mp/ssu067 (PMC4153440; doi:10.1093/mp/ssu067)
Supplement: Supplementary Data [file supp_7_9_1455__index.html]

OPT3 is a component of the iron-signaling network between leaves and roots and misregulation of OPT3 leads to an over-accumulation of cadmium in seeds — OPT3 Is a Component of the Iron-Signaling Network between Leaves and Roots and Misregulation of OPT3 Leads to an Over-Accumulation of Cadmium in Seeds — OPT3 Is a Component of the Iron-Signaling Network between Leaves and Roots and Misregulation of OPT3 Leads to an Over-Accumulation of Cadmium in Seeds — Supplementary Data 

# OPT3 Is a Component of the Iron-Signaling Network between Leaves and Roots and Misregulation of *OPT3* Leads to an Over-Accumulation of Cadmium in Seeds

## Supplementary Data

Data files

**Files in this Data Supplement:**

- Supplementary Data - Supplementary Data
