# Supplementary material for: OPT3 Is a Component of the Iron-Signaling Network between Leaves and Roots and Misregulation of OPT3 Leads to an Over-Accumulation of Cadmium in Seeds
Source: Mol Plant. 2014 May 31;7(9):1455–69. doi: 10.1093/mp/ssu067 (PMC4153440; doi:10.1093/mp/ssu067)
Supplement: Supplementary Data [file supp_ssu067_Supp_Figs_wlegends__MPlantR1_.pdf]

Cell Type Specific Arabidopsis eFP Browser

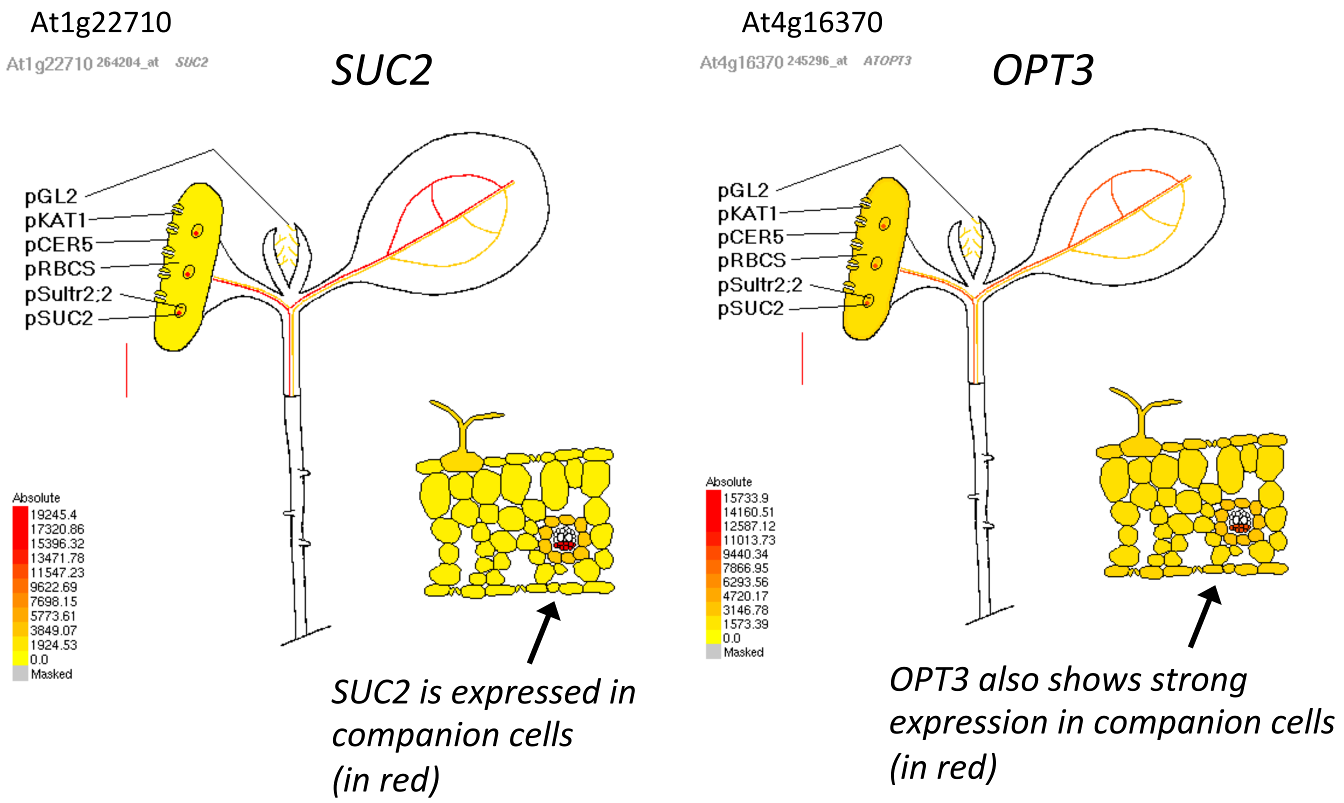

http://efp.ucr.edu/cgi-bin/absolute.cgi

Supplemental Figure 1: Cell type-specific translatome analyses show *OPT3* expression in the phloem (companion cells). Signal intensities of *OPT3* overlap with those of the phloem sucrose transporter *SUC2.*

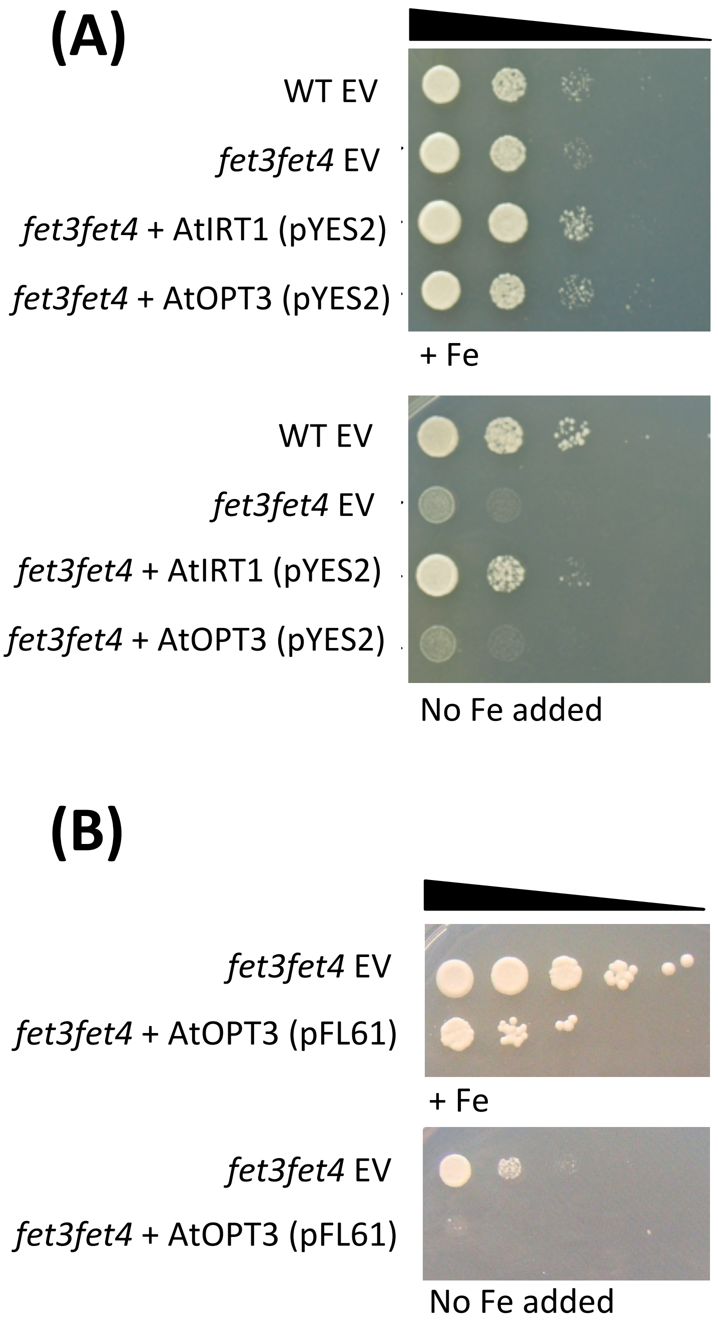

Supplemental Figure 2: *OPT3* does not rescue the growth of the *fet3fet4* yeast mutant on minimal media without supplemented Fe. **(A)** The yeast mutant *fet3fet4* can only grow on minimal media supplemented with Fe. Arabidopsis IRT1, but not OPT3, rescues the growth of *fet3fet4* on minimal media. Expression of IRT1 and OPT3 was driven by the GAL inducible promoter. **(B)** Expression of OPT3 driven by the constitutive promoter PGK also failed to rescue the growth of the *fet3fet4*. Note that *fet3fet4* yeast need to be iron starved prior to complementation tests to avoid growth of non-complementing strains.

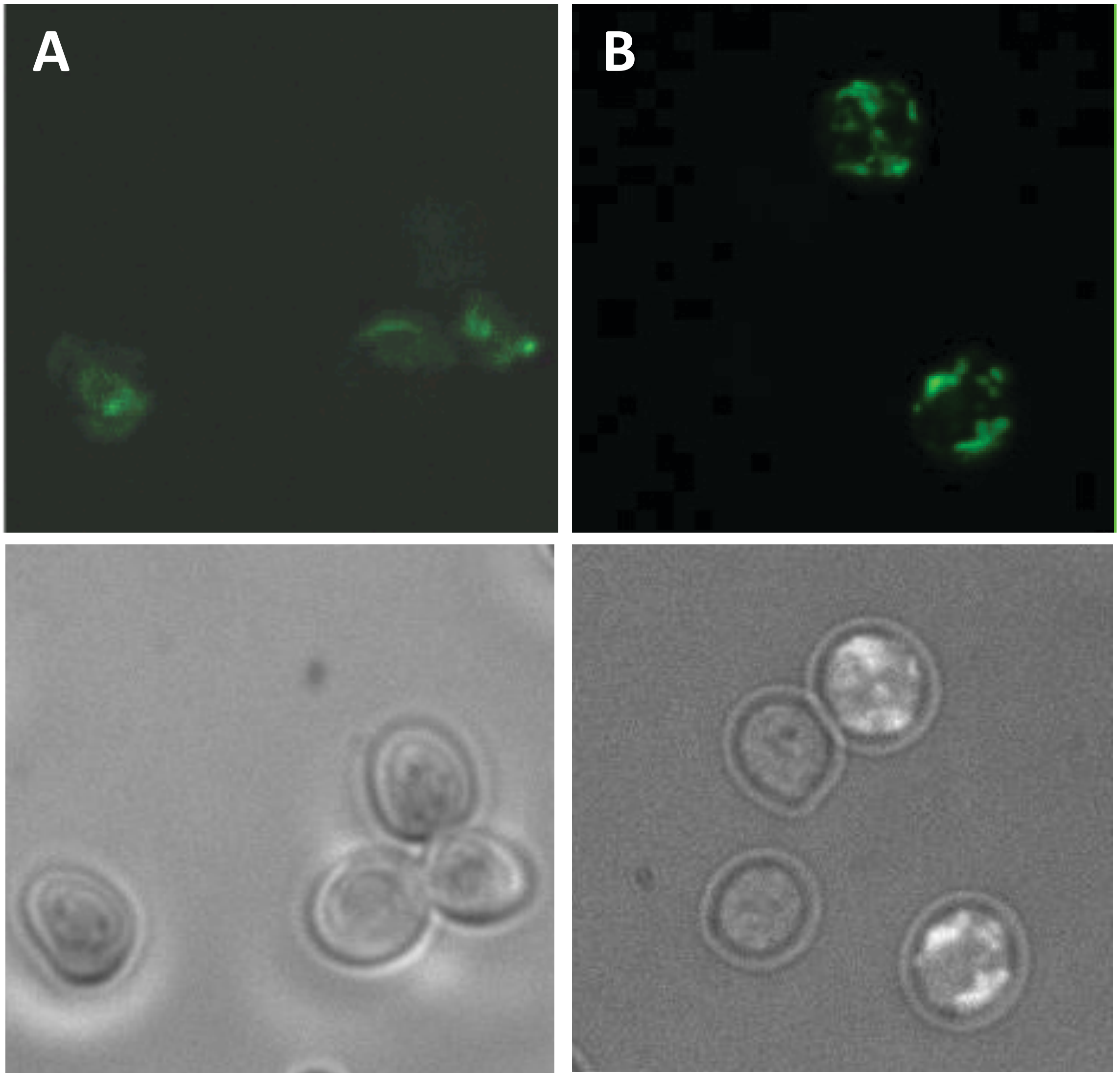

Supplemental Figure 3: OPT3 fails to transit out of the endoplasmic reticulum in *S. cerevisiae*. Brightfield and confocal microscopy of yeast expressing either (A) an N-terminal *YFP-OPT3* fusion or (B) a C-terminal *OPT3-YFP* fusion. Note the punctuate fluorescence throughout the cell. Notably, fluorescence is absent at the cell perimeter but present surrounding the nucleus, a typical hallmark of ER fluorescence in yeast. Bright-field images are shown below their respective fluorescence images.

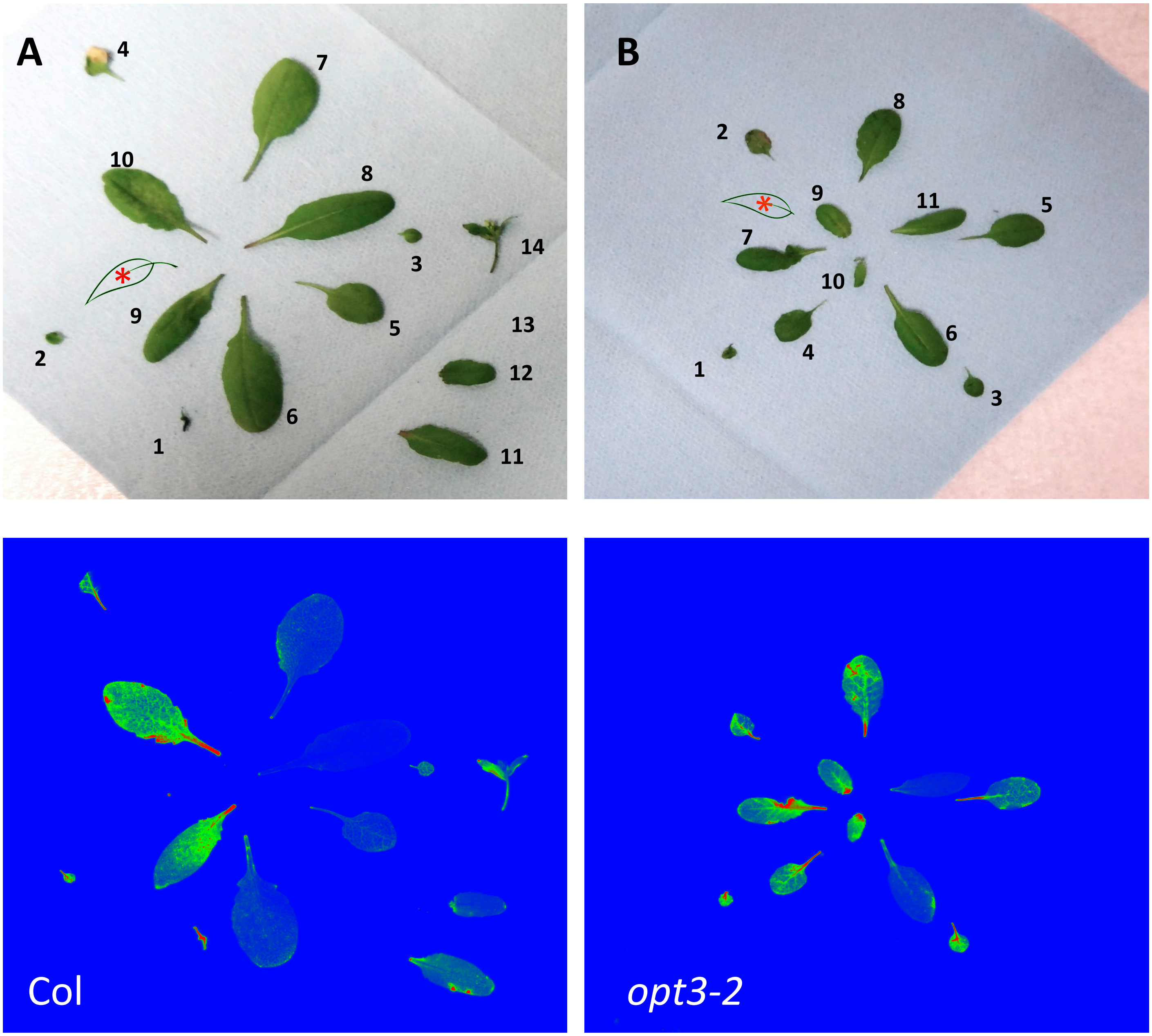

Supplemental Figure 4: Mobilization of ^{35}S-GSH between leaves is not impaired in *opt3-2*. ^{35}S-GSH was applied to a mature (A) wild-type or (B) *opt3-2* leaf and the distribution of ^{35}S-GSH was monitored after a 12 hr incubation period. The signal coming from ^{35}S-GSH detected with a PhosphorImager system shows that wild-type and *opt3-2* plants are able to mobilize ^{35}S-GSH to adjacent leaves (A and B, bottom panel).

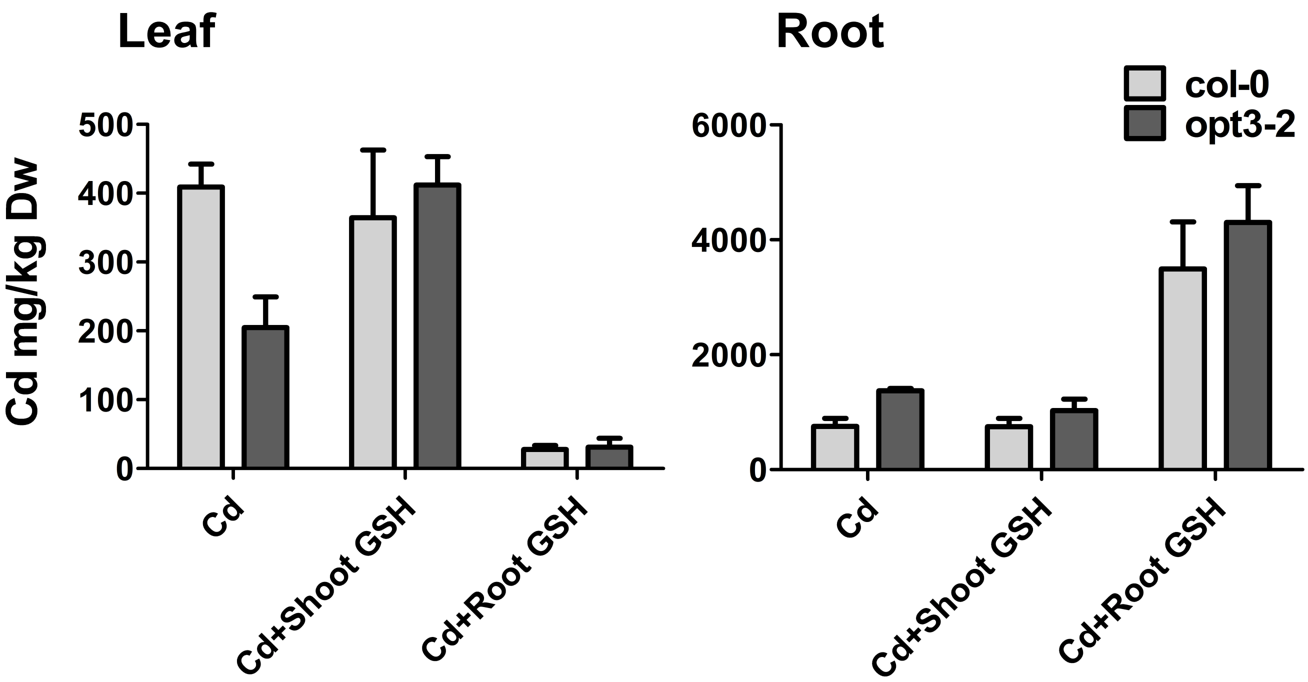

Supplemental Figure 5: Foliar (shoot) application of GSH to *opt3-2* restores Cd accumulation in leaves of *opt3-2* to wild-type levels. Cadmium content in leaves and roots was determined by ICP-OES. (A) In leaves, the Cd content in *opt3-2* was restored to wild type levels when applying foliar GSH (n=5). (B) In roots, Cd accumulation in *opt3-2* was not greatly affected by foliar (shoot) or root GSH application (n=4) compared to wild type in the presence and absence of GSH. Furthermore, exposure of roots to GSH (A, B) caused trapping of Cd in roots. Data represent mean ± SE.

(A)

(B)

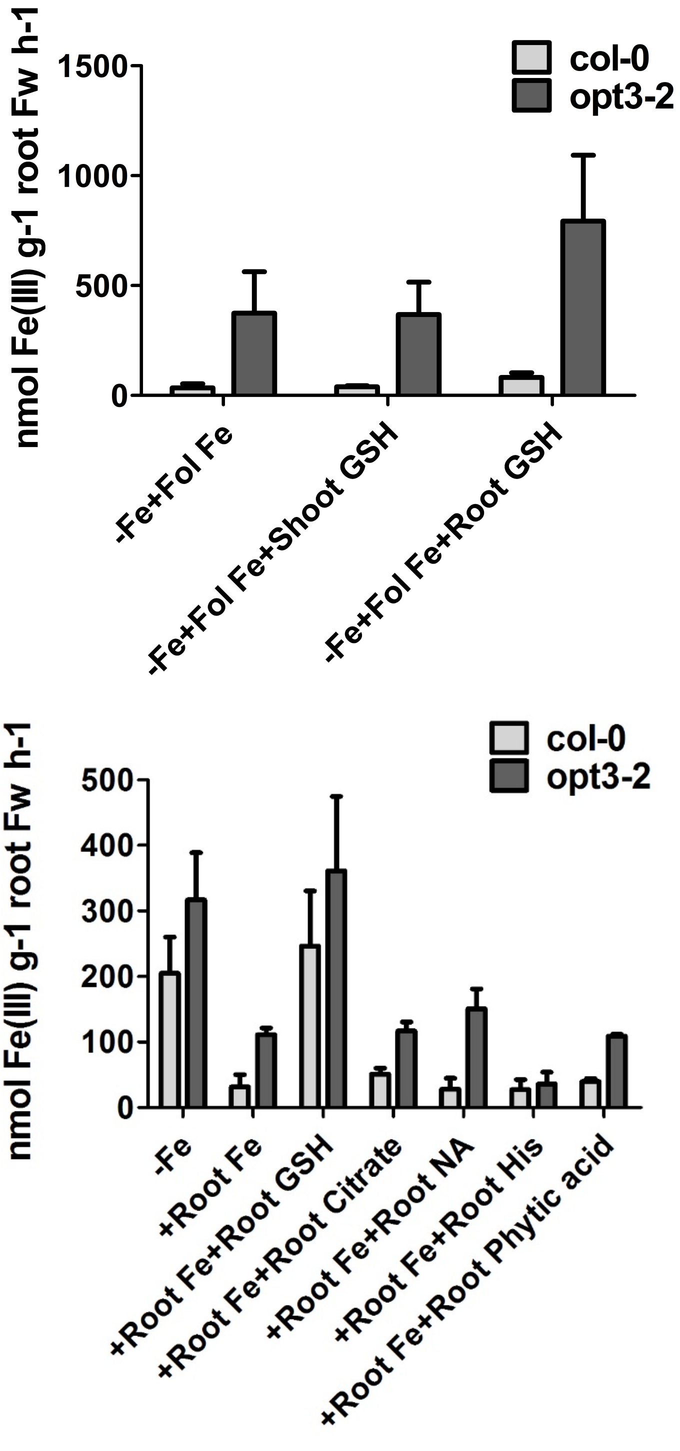

Supplemental Figure 6:: **(A)** Foliar application of GSH did not suppress the constitutive Fe-deficiency response in *opt3-2*. Root iron reductase activity of *opt3-2* remained much higher than wild type plants after foliar application of Fe. Applying GSH to the shoot did not suppress the high root reductase activity of *opt3-2*. GSH application to the root also did not inhibit root iron reductase activity. **(B)** Application of iron, alone or in combination with citrate or nicotianamine, to roots does not restore the activity of the Fe chelate reductase to wild type levels. Data represent mean ± SE (n=4-6).
